# Homœopathists vs. Homœopathy

**Published:** 1867-06

**Authors:** 


### *Homœopathists vs. Homœopathy.*

From a recent discussion which took place at the Société Médicale Homœopathique de France, it would seem that some of the leading homœopathists of Europe are abandoning not only the practice of administering infinitesimal doses (which, indeed, they have pretty generally done long since), but even their theoretical defence. M. Curie, son to the well known homœopathist of that name, having made the declaration in the Society that for his part he did not believe in the action of infinitesimal doses—or, at the very least, had his doubts respecting them—M. Leon Simon asked, in some dismay, "What then is to become of Homœopathic tradition? Are we to burn the books of our predecessors, and close our pharmacies? Are we to admit that we have been hitherto either charlatans or dupes! This is no question of mere doctrine, but one of fact, for we cannot have been following an erroneous course for sixty years without exhibiting complicity or stupidity." "Not so," replies M. Curie, "I can very well imagine the properties which may be developed in medicinal agents by infinitesimizing their division, but practically I have not met with them, and I attribute the good which results from their employment to the prevention of the perturbating action of allopathic procedures and leaving nature to her unimpeded action. What proof have you that your doses have effected cures, in the utter absence there is of all means of exact clinical comparison in the cases under your treatment?" M. Cretin observed that M. Curie did not deny the virtue of these infinitesimal doses, but remained under the influence of scientific doubt, ready to give way to conviction on the production of exact facts. For his own part, if, while accepting the law of *similia similibus*, he is to be expected to embrace all the contradictory opinions of Hahnemann and his followers, he must decline continuing to be a member of the society. M. Jousset observed that, in fact, homœopathy has entered on a new phase, the phase of friendly criticism. "In fact, if we look at our Materia Medica, we find a diffused heap of numberless symptoms, which are frequently contradictory or puerile, while the clinical facts that have been published in numerous instances are utterly destitute of the basis derived from diognosis, and exhibit the grossest ignorance. The comparison of the symptoms derived from the Materia Medica, and from poisonous substances, is sufficient, however, to convince us that Hahneman's observations are based on truth; while many clinical observations are due to skilful and honorable men. Then, again, our own daily practice convinces us, however difficult it may be to convey that conviction to others, that the success following the employment of infinitesimal doses cannot be explained as mere coincidences." M. Cretin commented on the disreputable character of much of homœopathic literature, and on the impossibility of getting the curative agencies so abundantly boasted of in books demonstrated in practice.—*Medical Times and Gazette.*

